# Geographic distribution and utilisation of CT and MRI services at public hospitals in Myanmar

**DOI:** 10.1186/s12913-020-05610-x

**Published:** 2020-08-12

**Authors:** Moe Khaing, Yu Mon Saw, Thet Mon Than, Aye Myat Mon, Su Myat Cho, Thu Nandar Saw, Tetsuyoshi Kariya, Eiko Yamamoto, Nobuyuki Hamajima

**Affiliations:** 1 Department of Healthcare Administration, Nagoya University Graduate School of Medicine, 65 Tsurumai-cho, Showa-ku, Nagoya, 466-8550 Japan; 2 Medical Care Division, Department of Medical Services, Ministry of Health and Sports, Nay Pyi Taw, Myanmar; 3 Nagoya University Asian Satellite Campuses Institute, Nagoya, Japan; 4 Department of Community and Global Health, The University of Tokyo, Tokyo, Japan

**Keywords:** Geographic distribution, Utilisation, CT, MRI, Myanmar

## Abstract

**Background:**

Diagnosis by computed tomography (CT) and magnetic resonance imaging (MRI) is important for patient care. However, the geographic distribution and utilisation of these machines in countries with limited resources, such as Myanmar, have not been sufficiently studied. Therefore, this study aims to identify the geographic distribution and utilisation of CT and MRI services at public hospitals in Myanmar.

**Methods:**

This nationwide, cross-sectional study was conducted at 43 public hospitals in Myanmar. Data were collected retrospectively using a prepared form from 1st January 2015 to 31st December 2017 at public hospitals in Myanmar. A descriptive analysis was performed to calculate the number of CT and MRI units per million population in each state and region of Myanmar. The distribution of CT and MRI units was assessed using the Lorenz curve and Gini coefficient, which are indicators of inequality in distribution.

**Results:**

In total, 45 CT and 14 MRI units had been installed in public hospitals in Myanmar by 2017. In total, 205,570 CT examinations and 18,981 MRI examinations have been performed within the study period. CT units per million population in 2017 varied from 0.30 in Rakhine State to 3.22 in Kayah State. However, MRI units were available only in public hospitals in five states/regions. The Gini coefficient for CT and MRI was 0.35 and 0.69, respectively. An upward trend in the utilisation rate of CT and MRI was also observed during the study period, especially among patients aged between 36 and 65 years.

**Conclusions:**

Throughout Myanmar, CT units were more equally distributed than MRI units. CT and MRI units were mostly concentrated in the Yangon and Mandalay Regions, where the population density is higher. The geographic distribution and utilisation rate of CT and MRI units varied among states, regions, and patients’ age group. However, the utilisation rates of CT and MRI increased annually in all states and regions during the review period. The Ministry of Health and Sports in Myanmar should consider the utilisation and population coverage of CT and MRI as an important factor when there will be procurement of those medical equipment in the future.

## Background

Healthcare is changing with the times, and current health systems utilise advanced technologies that continue to improve [1]. Imaging technology is of utmost importance for diagnosis and decision making in treatment [2]. These new technologies, such as computed tomography (CT), magnetic resonance imaging (MRI), and positron emission tomography–computed tomography (PET-CT), are replacing older ones in the diagnosis of diseases [2]. Among these, CT and MRI have been used increasingly due to their superior ability to differentiate the internal structures of the body and diagnose a wide range of different illnesses [1].

The enhanced ability of CT and MRI to differentiate tissues and organs is attractive to doctors as well as to patients, who would prefer easier access to high-tech imaging equipment [2]. CT has been used to differentiate abnormal body mass and changes such as tumours, infections, and cysts to produce three-dimensional images of organs [3]. CT is helpful in minimising the need for surgery and provides guidance in some procedures [4], and is widely used for the prevention and screening of diseases [5]. It can also be used in special procedures such as angiography, coronary CT angiography, and coronary CT calcium scans [3, 6]. MRI is more suitable for diagnosis related to soft tissues and the nervous system. MRI uses magnetic fields and radio waves to differentiate between tissues and organs, whereas CT uses the ionising radiation of X-rays. Notably, MRI takes more time and is noisy during imaging, and some conditions hinder MRI use, such as the presence of artificial heart valves and metal implants [7].

Imaging equipment is extremely intricate, and choices in equipment are broad. Advances in imaging technology have increased its diagnostic and treatment ability for a variety of diseases, ultimately improving the quality of healthcare [1, 2, 7, 8]. Modern technology has also impacted healthcare expenditure, largely because high-tech imaging equipment is costly [1, 2, 9]. Increasing the use of imaging equipment is a challenge for low-and middle-income countries; another issue is efficient utilisation, which is crucial for countries with a limited budget [1]. The utilisation of CT and MRI should be considered carefully because of the high costs involved; they should not be recommended as the first approach in detecting diseases without neurological deficits, such as bone fractures and lower back pain, that other imaging technologies such as X-rays and ultrasound can easily detect [8].

Although the utilisation of modern imaging technology is increasing, it is difficult to ascertain whether they are being used effectively [2]. Many countries have conducted studies on the utilisation patterns and distribution of imaging equipment to optimise effective allocation and reduce in appropriate usage [10–21]. The findings from these studies have been invaluable for controlling budget expenditure, especially in regions where funds are limited, such as in developing countries. A study in Taiwan reported significant variation in the distribution of imaging equipment in homogenous areas and unsatisfactory use of radiological services in other areas [22]. Iran also experienced a rapid spread of MRI utilisation through unplanned diffusion [1]. According to an assessment in China, the country had fewer CT and MRI units per million population in 2009 than most selected OECD countries; moreover, considerable inequity existed in terms of the characteristics and financing of CT and MRI units [14]. The distribution of imaging equipment may vary depending on the healthcare system, human resources, geography, transportation, as well as government policy [14].

Myanmar’s healthcare system has been changing with the political transition in recent years [23]. Healthcare delivery is funded by a pluralistic mix of public and private sources [23]. In 2012, the national maternal mortality ratio in Myanmar was 200 per 100,000 live births and the infant mortality rate was 41 [23]. The government’s contribution to the health sector increased dramatically in fiscal year 2012–2013, with a fourfold increase over the previous financial year’s budget [24]. In fiscal year 2014–2015, government health expenditure accounted for 0.99% of the gross domestic product [24]. Regarding health workforce, coverage was one medical doctor per 1477 population in the financial year 2015–2016 [24]. The Ministry of Health and Sports (MoHS) is the main provider of comprehensive healthcare in the entire country. In 2017, there were 1134 public hospitals under the MoHS and 68 hospitals under other ministries, mostly the Ministry of Defence [24]. Public hospitals under the MoHS are classified as station, township, district, state or regional, and national hospitals [25]. National hospitals include general hospitals with medical specialties, specialist hospitals, and teaching hospitals. National hospitals provide tertiary specialist healthcare, whereas most state or regional hospitals provide secondary healthcare services.

Geographic variation in healthcare challenges the basic principle of fair allocation of healthcare resources [16]. For a country like Myanmar, which is in the midst of political and economic transition, new insights or valuable information is important to inform policies and service delivery, including the delivery of healthcare services by, for instance, establishing new hospitals and health centres, as well as the procurement and allocation of high-tech equipment for healthcare facilities. With the healthcare budget increasing since 2012, MoHS has allocated funds for advanced technologies in public hospitals, such as CT, MRI, and PET-CT. The procurement of major equipment for public hospitals is overseen by the Department of Medical Services (DoMS) based on the demand of the hospitals and budget allowance. CT and MRI services are provided in public hospitals at a lower cost than in private hospitals, and some offer them for free.

As CT and MRI are a costly but important component of patient care, allocation should be fair among states and regions, and they should be utilised effectively. However, no information is available on the fairness of the distribution and nationwide availability of CT and MRI regarding the public sector in Myanmar. To the best of our knowledge, the utilisation rate of CT and MRI in Myanmar is also unknown. The government needs to be informed about equitable allocation and utilisation of CT and MRI for further procurement. Therefore, this study aimed to determine the geographic distribution of CT and MRI and their utilisation patterns along with population coverage.

## Methods

### Data collection

This nationwide, cross-sectional study was conducted to assess the distribution of CT and MRI equipment in public hospitals. The study included all public hospitals with functioning CT and MRI units: 40 hospitals (18 tertiary hospitals and 22 secondary hospitals) for CT and 13 hospitals (12 tertiary hospitals and one secondary hospital) for MRI. Some hospitals installed both CT and MRI. The data were collected retrospectively using a prepared form (Annex 1) from 1st January 2015 to 31st December 2017 at public hospitals and by accessing official reports of the DoMS and MoHS.

Data on the following were collected: types of CT and MRI, and patients’ age, sex, and area of the body examined. The locations of public hospitals with CT and MRI were obtained from the Procurement and Distribution Division of the DoMS. Hospitals enter details of CT and MRI examinations manually into their registers and record the patients’ name, registration number, age, sex, body part to be examined, findings, and charge. The data were extracted from these registers. The data entry was conducted by trained teams using the Microsoft Excel.

### Statistical analysis

The data were captured and a descriptive analysis was conducted to assess population coverage of CT and MRI. Projected population of Ministry of Immigration and Population, Myanmar, for the year 2017, 2016, and 2015 were used since they based upon population data from the 2014 Myanmar Census. Tables and figures were created using Microsoft Excel 2010. Descriptive analysis was used for the absolute numbers and numbers per million population of CT and MRI. The annual growth rates (AGRs) of CT and MRI were also calculated from 2015 or 2016 to 2017. The formula of AGR is as follows:

$$ \mathrm{AGR}=\sqrt[n]{\frac{B}{A}}-1, $$

where ***B*** is the quantity of CT or MRI in 2017, ***A*** is the quantity of CT or MRI in 2015 or 2016, and ***n*** represents the number of years [15].

For assessing the equity status of CT and MRI distribution, we used the Lorenz curve and Gini coefficient. The cumulative proportion of the population and the cumulative distribution of CT/MRI units were calculated to draw the Lorenz curve as the X and Y axes, respectively. All 15 states and regions were ranked by the number of CT or MRI units per million population. A diagonal line was drawn to mark perfect equality, and the equity status could be determined by the area between this perfect equality line and the Lorenz curve. The Gini coefficient was obtained by dividing this area by the area under the perfect equality line. The value of the Gini coefficient can be ranged between 0 and 1, and a higher value indicates greater inequality [13].

## Results

A total of 45 CT and 14 MRI units had been installed in public hospitals by the end of 2017. Most CT units were located in general hospitals, specialised orthopaedic and paediatric tertiary hospitals, and regional hospitals. Almost all of the MRI units were installed in tertiary hospitals, except one unit at the secondary Magway General Hospital. Table 1 describes the geographic distribution of CT and MRI units in 2017 by number and percentage. All states and regions had at least one CT unit. The Yangon and Mandalay Regions had 13 (28.9%) and seven (15.6%) CT units, respectively. MRI was available in only five states and regions: Yangon, Mandalay, Nay Pyi Taw, Magway, and Shan. In addition, 2016 and 2015 geographic distribution of CT and MRI units information are described in Annex 2 and 3, accordingly.

**Table 1:** Geographic distribution of CT and MRI at public hospitals in all states and regions of Myanmar (2017)

Region/State/Union Territory	CT				MRI			
	Tertiary hospital	Secondary hospital	Total	%	Tertiary hospital	Secondary hospital	Total	%
Kachin	–	2	2	4.4	–	–	–	–
Kayah	–	1	1	2.2	–	–	–	–
Kayin	–	1	1	2.2	–	–	–	–
Chin	–	1	1	2.2	–	–	–	–
Sagaing	–	2	2	4.4	–	–	–	–
Tanintharyi	–	2	2	4.4	–	–	–	–
Bago	–	3	3	6.7	–	–	–	–
Magway	1	2	3	6.7	–	1	1	7.1
Mandalay	6	1	7	15.6	3	–	3	21.4
Mon	–	1	1	2.2	–	–	–	–
Rakhine	–	1	1	2.2	–	–	–	–
Yangon	13	–	13	28.9	7	–	7	50.0
Shan	1	2	3	6.7	1	–	1	7.1
Ayeyarwady	–	2	2	4.4	–	–	–	–
Nay Pyi Taw	3	–	3	6.7	2	–	2	14.3
**Total**	**24**	**21**	**45**	**100**	**13**	**1**	**14**	**100**

### Distribution of CT and MRI units

**Fig. 1:**
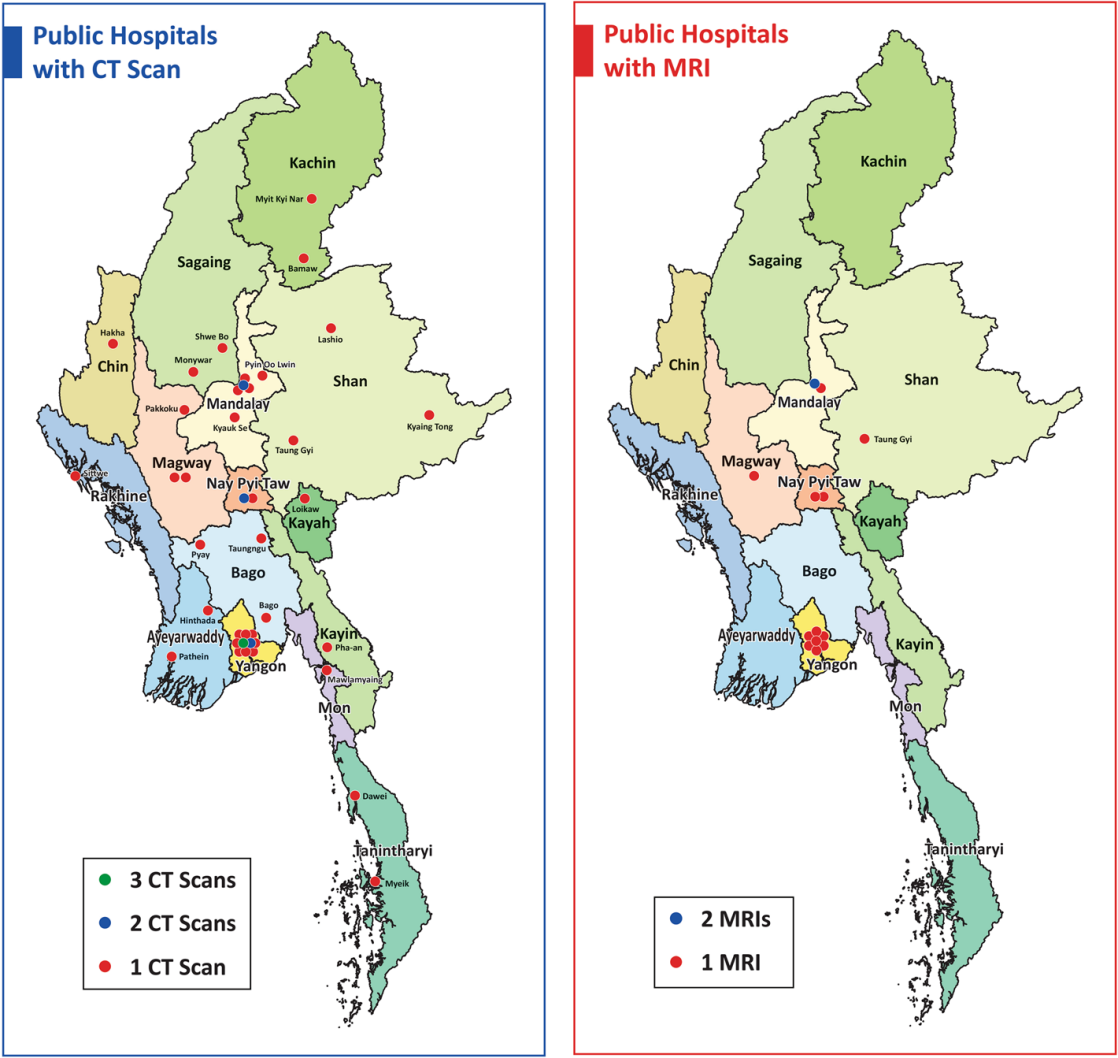
Geographic Distribution of CT and MRI at public hospitals in Myanmar (2017). Distribution of CT and MRI all over the country was described on country map of Myanmar separately. CT was available in all 15 states and regions of Myanmar while MRI was available in only five regions. There was one hospital (Yangon General Hospital) with three CT scans in Yangon Region, three hospitals (Mandalay General Hospital, Nay Pyi Taw General Hospital, Yangon Specialist Hospital) with two CT scans in Mandalay Region, Nay Pyi Taw Union Territory, and Yangon Region. There was also one hospital (Mandalay General Hospital) with two MRIs in Mandalay Region

Figure 1 shows the distribution of CT and MRI units at public hospitals in Myanmar. One tertiary hospital in Yangon Region (Yangon General Hospital) had three CT units, whereas three tertiary hospitals, Mandalay General Hospital (Mandalay Region), Nay Pyi Taw General Hospital (Nay Pyi Taw Union Territory), and Yangon Specialist Hospital (Yangon Region) had two CT units each. One tertiary hospital, Mandalay General Hospital (Mandalay Region) had two MRI units.

Table 2 shows the numbers and average AGR of CT and MRI units at public hospitals in all states and regions of Myanmar from 2015 or 2016 to 2017. The numbers of CT and MRI units both had been increasing during the period, while the number of MRI units grew faster than that of CT units. The numbers of CT and MRI units in Yangon and Mandalay Regions were larger than those in other states/regions, and so were the average AGR that were 33% and 14%, respectively for MRI. Average AGR for CT in Bago and Sagaing Regions were 44% and 41% respectively and were larger than those in other states/regions.

**Table 2:** Number and average growth rates of CT and MRI at public hospitals in all states and regions of Myanmar (2015–2017)

Region/State/Union Territory	CT				MRI			
	2015	2016	2017	AGR	2015	2016	2017	AGR^a^
Kachin	1	2	2	26%	–	–	–	–
Kayah	1	1	1	0%	–	–	–	–
Kayin	1	1	1	0%	–	–	–	–
Chin	0	1	1	0%	–	–	–	–
Sagaing	0	1	2	41%	–	–	–	–
Tanintharyi	2	2	2	0%	–	–	–	–
Bago	1	1	3	44%	–	–	–	–
Magway	2	2	3	14%	–	1	1	0%
Mandalay	5	6	7	12%	2	2	3	14%
Mon	1	1	1	0%	–	–	–	–
Rakhine	1	1	1	0%	–	–	–	–
Yangon	11	13	13	6%	3	6	7	33%
Shan	2	3	3	14%	–	1	1	0%
Ayeyarwady	1	2	2	26%	–	–	–	–
Nay Pyi Taw	2	2	3	14%	2	2	2	0%
**Total**	**31**	**39**	**45**	**13%**	**7**	**12**	**14**	**26%**

^a^*AGR* Annual growth rates

### Equity status of distribution of CT and MRI units

**Fig. 2:**
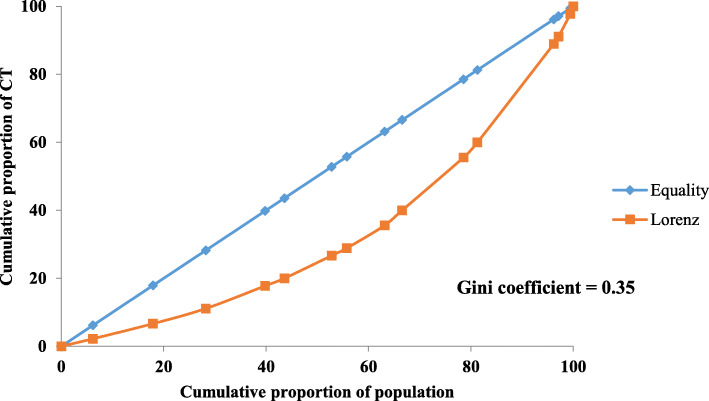
Lorenz Curve of CT at public hospitals in all states and regions of Myanmar (2017). Equity of distribution of CT among states and regions was assessed by Lorenz curve and Gini coefficient was calculated based on Lorenz curve. Lorenz curve showed disparity with equity line. Inequity of distribution of CT among states and regions was observed because Gini coefficient was relatively high

**Fig. 3:**
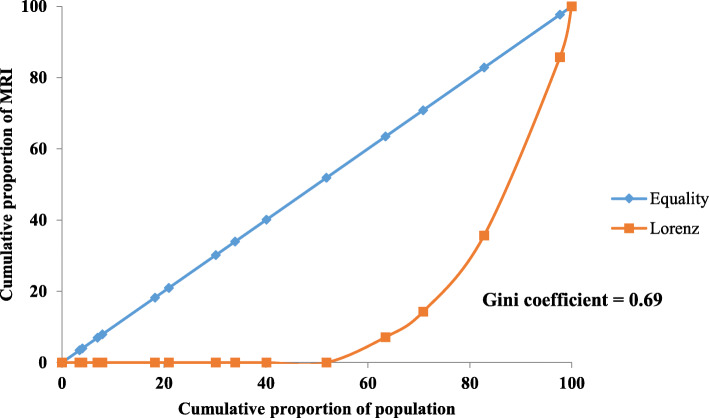
Lorenz Curve of MRI at public hospitals in all states and regions of Myanmar (2017). Equity of distribution of MRI among states and regions was assessed by Lorenz curve and Gini coefficient was calculated based on Lorenz curve. Distribution of MRI among states and regions was varied widely. Lorenz curve had wide disparity with equity line. Gini coefficient was high and approaching near to 1

The Lorenz curves for the distribution of CT and MRI units in Myanmar are shown in Figs. 2 and 3. The area between the diagonal line and the Lorenz curve depicting the distribution of MRI was larger (Gini coefficient 0.69), as MRI was not available in other states and regions except Yangon, Mandalay, Nay Pyi Taw, Magway, and Shan. The Lorenz curve for the distribution of CT units was separate from the diagonal line (Gini coefficient 0.35) because CT units were available in all states and regions of Myanmar. Furthermore, 2016 and 2015 the Lorenz curves for the distribution of CT and MRI units are described in Annex 4-7.

### Population coverage of CT and MRI

Table 3 shows the population coverage of CT and MRI units by state and region in 2017. The number of CT and MRI units per million population was, respectively, 0.84 and 0.26 for the whole country. The population coverage of CT was the highest in Kayah State, which had the lowest population. The second highest population coverage of CT was found in Nay Pyi Taw, the capital city of Myanmar. Nay Pyi Taw also had the highest population coverage for MRI. Low population coverage of CT was found in Rakhine, Ayeyarwaddy, and Sagaing compared with other states and regions. Moreover, 2016–2015 the population coverage of CT and MRI units by state and region are shown in Annex 8 and 9.

**Table 3:** Population coverage and utilisation of CT and MRI at public hospitals in all states and regions of Myanmar (2017)

Region/State/Union Territory	Population	CT				MRI			
		Number	Coverage per million population	No. of examination	Examination per 1000 population	Number	Coverage per million population	No. of examination	Examination per 1000 population
Kachin	1,829,848	2	1.09	1651	0.90	0	–	–	–
Kayah	310,214	1	3.22	1449	4.67	0	–	–	–
Kayin	1,593,053	1	0.63	2570	1.61	0	–	–	–
Chin	508,359	1	1.97	584	1.15	0	–	–	–
Sagaing	5,491,170	2	0.36	862	0.16	0	–	–	–
Tanintharyi	1,459,952	2	1.37	5059	3.47	0	–	–	–
Bago	4,918,821	3	0.61	2769	0.56	0	–	–	–
Magway	3,941,237	3	0.76	6475	1.64	1	0.25	148	0.04
Mandalay	6,389,390	7	1.10	20,386	3.19	3	0.47	2098	0.33
Mon	2,011,428	1	0.50	4797	2.38	0	–	–	–
Rakhine	3,300,041	1	0.30	1562	0.47	0	–	–	–
Yangon	7,936,637	13	1.64	129,445	16.31	7	0.88	13,285	1.67
Shan	6,118,689	3	0.48	3363	0.54	1	0.16	163	0.03
Ayeyarwady	6,271,070	2	0.32	2830	0.45	0	–	–	–
Nay Pyi Taw	1,238,039	3	2.42	21,768	17.58	2	1.62	3287	2.66
**Total**	**53,387,948**	**45**	**0.84**	**205,570**	**3.85**	**14**	**0.26**	**18,981**	**0.36**

Table 4 shows the changing trend of the numbers of CT and MRI units per million population in states/regions of Myanmar from 2015 or 2016 to 2017. Numbers in Kayah and Nay Pyi Taw were higher than other states/regions during the period. Average AGR were higher in Bago and Sagaing Regions for CT units. Average AGR was 31% in Yangon Region for MRI and Yangon Region grew the fastest. The number of CT per million population in Bago Region grew the fastest.

**Table 4:** Number of CT and MRI per million population and average growth rates in all states and regions of Myanmar (2015–2017)

Region/State/Union Territory	CT				MRI			
	2015	2016	2017	AGR^a^	2015	2016	2017	AGR^a^
Kachin	0.57	1.11	1.09	24%	–	–	–	–
Kayah	3.37	3.29	3.22	-2%	–	–	–	–
Kayin	0.63	0.63	0.63	0%	–	–	–	–
Chin	0	1.99	1.96	−1%	–	–	–	–
Sagaing	0	0.18	0.36	41%	–	–	–	–
Tanintharyi	1.39	1.38	1.37	0%	–	–	–	–
Bago	0.20	0.20	0.60	44%	–	–	–	–
Magway	0.51	0.51	0.76	14%	–	0.25	0.25	0%
Mandalay	0.79	0.95	1.09	11%	0.32	0.32	0.47	14%
Mon	0.49	0.49	0.49	0%	–	–	–	–
Rakhine	0.31	0.31	0.30	-1%	–	–	–	–
Yangon	1.44	1.67	1.64	4%	0.39	0.77	0.88	31%
Shan	0.33	0.49	0.48	13%	–	0.16	0.16	0%
Ayeyarwady	0.16	0.32	0.32	26%	–	–	–	–
Nay Pyi Taw	1.67	1.64	2.42	13%	1.67	1.64	1.62	-1%
**Total**	**0.59**	**0.73**	**0.84**	**12%**	**0.13**	**0.23**	**0.26**	**26%**

^a^*AGR* Annual growth rates

### Types and yearly installation status of CT and MRI units in public hospitals

Table 5 shows the types of CT and MRI units by state and region. Most of the CT units were 16-slice types, and 64-slice CT units were available only in the four major regions of Yangon, Mandalay, Nay Pyi Taw, and Magway. Regarding MRI types, nine of the 14 MRI units were of strength 1.5 Tesla and above. By the end of 2015, 31 CT and seven MRI units had been installed, and eight CT (seven 16-slice and one 64-slice machines) and six CT (four 16-slice and two 64-slice machines) units were installed in 2016 and 2017, respectively. The installed CT in 2016 were found in Kachin, Chin, Sagaing, Mandalay, Yangon, Shan, and Ayeyarwady Regions/States and in 2017 were found in Sagaing, Bago, Magway, Mandalay, and Nay Pyi Taw. Five MRI (two 0.4 Tesla machines, two 1.5 Tesla machines, and one 3.0 Tesla machine) and two MRI (1.5 Tesla machines) units were installed in 2016 and 2017, respectively.

**Table 5:** Types of CT and MRI at public hospitals in all states and regions of Myanmar (2017)

Region/State/Union Territory	Types of CT Scan				Types of MRI				
	64 Slices	16 Slices	8 Slices	Total	3.0 Tesla	1.5 Tesla	0.4 Tesla	0.3 Tesla	Total
Kachin	–	2	–	2	–	–	–	–	0
Kayah	–	1	–	1	–	–	–	–	0
Kayin	–	1	–	1	–	–	–	–	0
Chin	–	1	–	1	–	–	–	–	0
Sagaing	–	2	–	2	–	–	–	–	0
Tanintharyi	–	2	–	2	–	–	–	–	0
Bago	–	3	–	3	–	–	–	–	0
Magway	1	2	–	3	–	–	1	–	1
Mandalay	1	5	1	7	–	2	–	1	3
Mon	–	1	–	1	–	–	–	–	0
Rakhine	–	1	–	1	–	–	–	–	0
Yangon	6	7	–	13	1	5	1	–	7
Shan	–	3	–	3	–	1	–	–	1
Ayeyarwady	–	2	–	2	–	–	–	–	0
Nay Pyi Taw	2	1	–	3	–	–	2	–	2
**Total**	**10**	**34**	**1**	**45**	**1**	**8**	**4**	**1**	**14**

### Utilisation pattern of CT and MRI units

Table 3 also shows the number of examinations and examinations per 1000 population. Examinations per 1000 population were the highest in Nay Pyi Taw, followed by Yangon, for both CT and MRI examinations. CT utilisation was the lowest (0.16) in the Sagaing Region. CT and MRI examinations per 1000 population were lower in Mandalay than in Nay Pyi Taw and Yangon.

The total number of CT examinations was extremely high in the Yangon Region compared with other regions, while the number of CT examinations was higher in the Nay Pyi Taw Union Territory than in the Mandalay Region. The other 12 states or regions accounted for 35,625 CT examinations—less than a third (27.0%) of the examinations in the Yangon Region. The number of MRI examinations was also the highest in the Yangon Region; the Nay Pyi Taw Union Territory had more MRI examinations than the Mandalay Region. The Yangon Region accounted for 62.9% (*n* = 129,445) of the total number of CT examinations (*n* = 205,570) and 69.9% (*n* = 13,285) of the total number of MRI examinations (*n* = 18,981) within the study period. Regarding the patients who received CT examinations, more than a third (34.5% in 2015, 34.3% in 2016 and 35.1% in 2017) were aged between 46 and 65 years. However, younger patients (aged 36 to 55 years) received MRI examinations. Male patients utilised CT services more often than female patients (59.7% vs. 40.3% in 2015; 58.5% vs. 41.4% in 2016; 57.3% vs. 42.7% in 2017). Utilisation of MRI services showed only a small difference by gender since male patients utilised more MRI services in 2015 (52.6% vs. 47.4%), female patients showed higher utilisation in 2016 and 2017 (49.8% vs 50.2% in 2016, and 46% vs. 54% in 2017) [Table 6].

**Table 6:** Age distribution and gender of patients who utilized CT and MRI at public hospitals in Myanmar (2015–2017)

	2015				2016				2017			
	^a^CT		^b^MRI		^a^CT		^b^MRI		^a^CT		^b^MRI	
	N	%	N	%	N	%	N	%	N	%	N	%
**Age**												
≤ 15	2420	10.1	130	6.9	4080	11.8	317	7.2	3193	7.7	432	8.1
16–25	2387	10.0	220	11.7	3554	10.3	453	10.4	4320	10.4	538	10.1
26–35	2731	11.4	271	14.5	3881	11.3	711	16.3	4883	11.8	874	16.3
36–45	3194	13.3	380	20.3	4604	13.4	824	18.8	5783	13.9	903	16.9
46–55	4092	17.1	408	21.8	5685	16.5	914	20.9	6989	16.9	1102	20.6
56–65	4172	17.4	282	15.0	6145	17.8	696	15.9	7549	18.2	887	16.6
66–75	2800	11.7	124	6.6	3996	11.6	328	7.5	5541	13.4	447	8.4
≥ 76	1672	7.0	48	2.6	2284	6.6	121	2.8	3012	7.3	148	2.8
Unknown	499	2.1	11	0.6	250	0.7	9	0.2	205	0.5	15	0.3
**Total**	**23,967**	**100**	**1874**	**100**	**34,479**	**100**	**4373**	**100**	**41,475**	**100**	**5346**	**100**
**Gender**												
Male	14,306	59.7	986	52.6	20,169	58.5	2176	49.8	23,761	57.3	2457	46.0
Female	9660	40.3	888	47.4	14,284	41.4	2197	50.2	17,701	42.7	2889	54.0
Unknown	1	0.0			26	0.1			13	0.0		
**Total**	**23,967**	**100**	**1874**	**100**	**34,479**	**100**	**4373**	**100**	**41,475**	**100**	**5346**	**100**

^a^ Age and gender data of CT scan examination were not available for Yangon General Hospital and Mandalay General Hospital

^b^ Age and gender data of MRI Examination were not available for Yangon General Hospital

Tables 7 and 8 describe the utilisation of CT and MRI by examination area (i.e. body part) from 2015 to 2017. CT examinations were mainly utilised for cranial/head examinations (60.0% in 2015, 60.7% in 2016, and 58.8% in 2017). The CT examinations of the abdomen and thorax regions (with or without other areas) accounted for the second and third highest number of examinations. Between 2015 and 2017, MRI was utilised mainly for spinal examinations (53.4 to 59.1%), with most examinations focused on the lumbar spine region (28.4% in 2015, 35.4% in 2016, and 30.9% in 2017). However, this data did not include the Yangon General Hospital (both CT and MRI) and Mandalay General Hospital (CT only), because although utilisation was extremely high in these hospitals, accurate data were not available. These hospitals’ records were also missing data for patients’ age, sex, and body part examined.

**Table 7:** Utilisation of CT by examination region at public hospitals^b^ in Myanmar (2015–2017)

Region of body examined	2015		2016		2017	
	N	%	N	%	N	%
**CT examination**						
^a^Cranial/Head	14,388	60.0	20,935	60.7	24,384	58.8
Head & neck	356	1.5	466	1.4	479	1.2
Head & other region	428	1.8	321	0.9	822	2.0
Whole spine	11	0.0	11	0.0	14	0.0
Cervical spine	112	0.5	140	0.4	212	0.5
Thoracic spine &Thoraco-lumbar spine	79	0.3	160	0.5	221	0.5
Lumbar spine &Lumbo-sacral spine	65	0.3	134	0.4	257	0.6
Abdominal and/or pelvic	4159	17.4	6160	17.9	7130	17.2
Thorax and other region	3485	14.5	4912	14.2	5821	14.0
Body (thorax and abdomen)	60	0.3	185	0.5	343	0.8
Whole body	1	0.0	3	0.0	1	0.0
Pelvis	24	0.1	73	0.2	88	0.2
Angiogram	93	0.4	117	0.3	202	0.5
Limbs/Joints	77	0.3	111	0.3	235	0.6
Other	629	2.6	751	2.2	1266	3.1
**Total**	**23,967**	**100**	**34,479**	**100**	**41,475**	**100**

^a^CT examination of cranial/head region accounted (*n* = 25,252, 74.89%) of all examination (*n* = 33,720) in regional level hospitals

^b^Data were not available for Yangon General Hospital and Mandalay General Hospital

**Table 8:** Utilisation of MRI by examination region at public hospitals^a^ in Myanmar (2015–2017)

Region of body examined	2015		2016		2017	
	N	%	N	%	N	%
Brain/Head	529	28.2	983	22.5	1486	27.8
Whole spine	12	0.6	67	1.5	18	0.3
Cervical spine	312	16.6	499	11.4	748	14.0
Thoracic spine &Thoraco-lumbar spine	211	11.3	473	10.8	440	8.2
Lumbar spine	533	28.4	1546	35.4	1650	30.9
Abdominal and/or pelvic	35	1.9	145	3.3	262	4.9
Pelvic	49	2.6	69	1.6	78	1.5
Both hips	11	0.6	103	2.4	79	1.5
Thorax	43	2.3	10	0.2	30	0.6
Limbs/Joints	77	4.1	292	6.7	304	5.7
MRCP	39	2.1	51	1.2	78	1.5
MRV/MRA	3	0.2	10	0.2	19	0.4
Other	20	1.1	125	2.9	154	2.9
**Total**	**1874**	**100**	**4373**	**100**	**5346**	**100**

^a^Data were not available for Yangon General Hospital; *MRCP* Magnetic resonance cholangiopancreatography, *MRV* Magnetic resonance venography, *MRA* Magnetic resonance angiography

We also compared the performance of CT and MRI examinations between tertiary and secondary hospitals; the results are shown in Table 9. A large gap was found between tertiary and secondary hospitals: 171,850 CT examinations were recorded at tertiary hospitals and only 33,720 CT examinations at secondary hospitals. The average number of examinations per CT unit in tertiary hospitals was 2162.9, 2551.2, and 3109.5 in 2015, 2016, and 2017, respectively; and the average number of CT examinations in secondary hospitals was 627.0, 696.1, and 683.9 in 2015, 2016, and 2017, respectively. Tertiary hospitals had 13 MRI units available, and only one was available at a secondary hospital; the number of examinations at the secondary hospital accounted for only 0.8% of the total.

**Table 9:** Performance of CT and MRI in Myanmar by level of public hospitals (2015–2017)

Level of hospitals	CT			MRI		
	Cumulative number	Number of examination	Number examinations per CT	Cumulative number	Number of examination	Number of examinations per MRI
**Tertiary hospitals**						
2015	19	41,096	2162.94	7	6120	874.29
2016	22	56,127	2551.22	11	6057	550.64
2017	24	74,627	3109.45	13	6656	512.00
Sub-total	24	171,850 (83.6%)	7160.41	13	18,833 (99.2%)	1488.69
**Secondary hospitals**						
2015	12	7524	627.00			
2016	17	11,834	696.11	1	25	25.00
2017	21	14,362	683.90	1	123	123.00
Sub-total	21	33,720 (16.4%)	1584.29	1	148 (0.8%)	148.00
**Grand Total**	**45**	**205,570 (100%)**		**14**	**18,981 (100%)**	

## Discussion

This study is the first to provide a nationwide assessment of the distribution and utilisation of CT and MRI equipment in the public sector. Most CT and MRI units were located in the largest cities, where tertiary hospitals and teaching hospitals were located. Out of 39 state/regional level hospitals under DoMS, 22 had installed CT units during the study period. Utilisation was high in states and regions with a higher number of CT and MRI units. Middle-aged people utilised high-tech imaging services more often, and mostly for examinations of the skull.

### Distribution and equity status of CT and MRI units

The installation of CT units in state and regional hospitals and in national hospitals was prioritised, and therefore, CT services are accessible in all states and regions. Most CT and MRI units were concentrated in the largest cities—Yangon, Mandalay, and Nay Pyi Taw. These cities are located in central Myanmar, where the population density is the highest and transportation services are also relatively good. This finding was consistent with that of a study on neuro-imaging facilities in Pakistan, which found that most CT and MRI units are located in major cities such as Karachi [26]. In Myanmar, tertiary hospitals with different areas of specialisation are located in areas that can be easily accessed from all over the country. State/regional hospitals are also located in these major regional cities and serve as referral hospitals for district, township, and station hospitals as well as other health facilities. The government prioritised the installation of high-tech diagnostic and treatment facilities in these tertiary and state/regional hospitals.

The Lorenz curve and Gini coefficient of MRI distribution by state and region showed a wide disparity. MRI was available in only five cities (Yangon, Mandalay, Nay Pyi Taw, Magway, and Taunggyi). Magway and Taunggyi both have teaching hospitals for university-level education and are both easily accessible from the Eastern and Western parts of Myanmar. Yangon General Hospital had three CT units, whereas Mandalay General Hospital and Nay Pyi Taw General Hospital had two each. This allocation was because these hospitals were the largest tertiary hospitals and had the highest hospital utilisation rate. Although the distribution of CT units by state and region was more equitable compared with that of MRI units, the disparity was nonetheless high between the states and regions with big cities and those without. The availability of high-tech equipment tends to reflect the development of a country, its states, and regions [14, 15]. Thus, the levels of development across Myanmar’s states and regions may be different.

Equity and accessibility are important considerations for healthcare services. Primary and advanced healthcare services should be fairly accessible throughout a country. Especially in a country like Myanmar, which has a varied topography, the provision of these services should be decided based on geographic area in addition to population density. However, other resources such as specialists, technicians, technical skills, and electricity supply are also essential components of high-tech diagnostic services. Thus, the allocation of medical resources should also be reasonable. The government needs to improve the competence of healthcare personnel and meet the basic needs of people living in more remote areas to ensure fair distribution of CT and MRI services.

Population density is an important consideration in CT and MRI allocation in Myanmar. The Myanmar government procured CT units for all states and regions particularly based on the location of state/regional hospitals. Population coverage of CT in states and regions with large populations, such as Rakhine, Sagaing, and Ayarwaddy, was lower than in other states and regions. Kayah State, which had the lowest population density, had good coverage. The biggest cities in terms of population density, such as Yangon, Mandalay, and Nay Pyi Taw, also had higher population coverage because they had more tertiary-level hospitals, so patients who needed advanced care were referred to these hospitals. Meanwhile, as MRI services were available only in five cities, the overall population coverage was low. Nonetheless, these cities were located in central Myanmar, which has a good transportation network and adequately qualified and relevant healthcare professionals.

In 2015, high-tech equipment per million population in cities in Guangxi, Southern China, ranged from 4.19 to 9.02 for CT and from 0.46 to 3.57 for MRI [10]. Guangxi is an autonomous region of the People’s Republic of China, located in Southern China. It is one of five ethic minority regions in China. Guangxi included 14 prefectural-level cities and had total population of 47,960,000 in 2015. Myanmar is a developing country with 135 ethnic groups, one Union Territory, and 14 states/regions with the population of 51,486,253. Guangxi, its total population and their administrative regions were comparable with Myanmar. Compared with these cities, the population coverage of CT and MRI services in Myanmar was low. Overall CT and MRI units per million population in Myanmar were lower than those in cities in Southern China in a previous study (lowest: 6.0 for CT and 1.3 for MRI) and with selected OECD countries (lowest: 7.4 for CT and 1.7 for MRI) in 2009 [14]. As a developing country, Myanmar does not need as high population coverage as in developed countries, but the coverage of CT services should be improved in states and regions that do not have easy access to large cities with high-quality CT and MRI services. For improvement of CT services, hospitals need to be upgraded with the necessary medical equipment and trained personnel. To improve population coverage, expanding CT and MRI services through the private sector is a feasible solution.

### Installation and types of CT and MRI units

Before 2012, CT and MRI services were only available in a few tertiary hospitals. According to the equipment and its usage register of hospitals, the earliest CT installation in Myanmar took place in 2007 at Pyin Oo Lwin General Hospital, whereas the first MRI unit was procured in fiscal year 2006–2007 for the Nay Pyi Taw General Hospital. We found that 31 out of 45 CT units and seven out of 14 MRI units had already been installed by 2015. The rate of CT and MRI installation decreased between 2016 and 2017. A reason for this decline was that CT procurement had reached peak in fiscal year 2012–2013, when the government budget for the health sector was increased. Fewer MRI units were installed, and their installation occurred later than large-scale CT installation.

Most CT machines were the 16-slice type and only a few were the 64-slice type. Compared with 2016, more 64-slice CT units were installed in 2017. Similarly, most MRI units were of strength 1.5 Tesla, and low-magnetic MRI machines (0.4 and 0.3 Tesla) were not installed after 2017. The procurement patterns of high-tech machines by the MoHS seemed to have shifted to meet the demands of advanced healthcare.

According to official reports of the Private Health Division of the DoMS, the private sector contributed almost half (46.0%) of all CT and MRI units, but private sector installations only covered eight states and regions for CT and only three regions for MRI. The contribution of Myanmar’s private sector in 2016 was less than that in Southeast Nigeria: Southeast Nigeria’s private hospitals owned 19 (67.1%) of the 28 CT machines in the region [20]. However, the earliest CT installation in Southeast Nigeria was in 1998, with most current units installed before 2010 [20]. CT units vary from one-slice to 32-slice types. In Myanmar, CT installations occurred later than in Southeast Nigeria, and therefore, had more advanced CT units installed. Japan, the country with the highest number of CT and MRI units per population in the world, had 12,945 CT and 5990 MRI units in 2011 [18, 27].

### Utilisation pattern of CT and MRI services

Utilisation was also high in states and regions with a higher number of CT and MRI units. This finding is in line with a previous study that found a direct correlation between the number of CT machines and the number of CT examinations [28]. This finding may be due to the presence of referral hospitals (i.e. tertiary hospitals) in these regions. Notably, Kayah State had a relatively high CT examination rate per 1000 population, which may be due to the increased utilisation of hospital services or higher utilisation by doctors. Regarding MRI utilisation, Nay Pyi Taw had more examinations per 1000 population than Yangon and Mandalay because Nay Pyi Taw is a new capital city and receives referral patients from central and upper Myanmar. Yangon Region accounted for a large percentage of the total CT and MRI utilisation rate—almost a third (29.0%). One implication is that Yangon Region had a heavier workload than any other region. Further policy planning of CT and MRI procurement should also consider the lifespan of machines and workload of health staff.

Although older and female patients are more likely to use health resources [29], CT and MRI utilisation was higher for middle-aged people and nearly equal between male and female patients in the present study. Thus, CT and MRI services were utilised more by the working age group. Regarding body regions subjected to CT examinations, more than half were carried out on the cranial/head region. This finding was consistent with the fact that injuries were found among the single leading cause of morbidity and mortality in Myanmar. In the Health in Myanmar (2014) report, injuries in specified, unspecified, and multiple body regions accounted for the leading cause of morbidity in 2012: intracranial haemorrhage, intracranial injury, and stroke were listed in the top 15 leading causes for that year [30]. MRI for different spinal and brain examinations was common [8, 31].

As such, CT and MRI were mostly used to diagnose abnormalities in the head and spine. Other advanced examinations were also performed, such as angiograms by CT and cholangiopancreatography by MRI. To assess the effective utilisation of CT and MRI, the indications and outcomes of inpatients need to be examined. This study did not assess such information, and further detailed studies are recommended.

When we compared the performance of CT examinations between tertiary and secondary hospitals, the former accounted for 83.6% of CT examinations, although the number of CT machines in the two hospital types was nearly the same. Moreover, secondary hospitals mostly performed CT examinations for assessing the head region. The limited capacity of secondary hospitals in terms of human resources and machine type leads to a large gap of utilisation between secondary and tertiary hospitals. Utilisation of tertiary hospitals was also very high because of patients’ preferences as a consequence of weak referral policies and more advanced facilities in tertiary hospitals. However, further studies should assess equity in utilisation and effective use. The workload of CT and MRI units in tertiary hospitals should be considered to extend the lifespan of these machines, which have maximum capacity only for a defined period.

### Strengths and limitations of the study

The main strength of this study is its use of nationwide data regarding the installation status of CT and MRI machines. As such, it could demonstrate the utilisation pattern of these machines. Lorenz curves were drawn and Gini coefficients were calculated to assess equity and distribution. However, the following limitations should be noted. This study did not include data on patients’ age and body part examined in CT at the Yangon General Hospital and Mandalay General Hospital because the information was not available; both hospitals handled very large numbers of examinations and used a manual record system for CT and MRI examinations. Moreover, difficulties were encountered during data entry as details of CT and MRI examinations were recorded manually in registers, with missing information for patients’ age and sex that showed up in the analysis as unknown entities. Furthermore, detailed information on the diagnoses and outcomes of inpatients could not be included.

## Conclusion

The CT units were more equally distributed than the MRI units in Myanmar. The CT and MRI units were mostly concentrated in the Yangon and Mandalay Regions, where the population density is extremely high. The geographic distribution and utilisation rate of CT and MRI varied among states, regions, and patients’ age groups. However, the utilisation rates of CT and MRI increased annually in all states/regions during the study period. Utilisation and population coverage of CT and MRI should be considered as important factor when there will be the future procurement of CT and MRI. Moreover, the lifespan, maintenance, and workload of CT and MRI machines should be considered in the planning of future installations. Hospitals in peripheral states and regions should be developed to provide more equitable health services. To improve equity of CT and MRI services among all states and regions and to reduce workload in tertiary hospitals, the private sector should be encouraged to provide CT and MRI services across Myanmar. Further studies to assess the cost effectiveness and effective utilisation of CT and MRI machines will be needed.

## Supplementary information

**Additional file 1: Annex 1.** Prepared form. **Annex 2.** Geographic Distribution of CT and MRI at public hospitals in all states and regions of Myanmar (2016). **Annex 3.** Geographic Distribution of CT and MRI at public hospitals in all states and regions of Myanmar (2015). **Annex 4.** Lorenz Curve of CT at public hospitals in all states and regions of Myanmar (2016). **Annex 5.** Lorenz Curve of CT at public hospitals in all states and regions of Myanmar (2015). **Annex 6.** Lorenz Curve of MRI at public hospitals in all states and regions of Myanmar (2016). **Annex 7.** Lorenz Curve of MRI at public hospitals in all states and regions of Myanmar (2015). **Annex 8.** Population coverage and utilisation of CT and MRI at public hospitals in all states and regions of Myanmar (2016). **Annex 9.** Population coverage and utilisation of CT and MRI at public hospitals in all states and regions of Myanmar (2015).

## Data Availability

The data that support the findings of this study are available from upon request as a requirement of the Institutional Review Board, Ministry of Health and Sports, Myanmar for researchers who meet the criteria for access to confidential data. Researchers who would like to access to the data must contact Medical Care Division, Department of Medical Services, Office no. 4, Ministry of Health and Sports, Ministry Zone, Nay Pyi Taw 15011, Myanmar. Tel& Fax: 95–67-3411002. Email: medicalcare@mohs.gov.mm

## Abbreviations

CT: Computed tomography
MRI: Magnetic resonance imaging
PET-CT: Positron emission tomography – computed tomography
CAT: Computerized axial tomography
OECD: Organization for Economic Co-operation and Development
MoHS: Ministry of Health and Sports
DoMS: Department of Medical Services
AGR: Annual growth rate
MRCP: Magnetic resonance cholangiopancreatography
