# Supplementary material for: Geographic distribution and utilisation of CT and MRI services at public hospitals in Myanmar
Source: BMC Health Serv Res. 2020 Aug 12;20:742. doi: 10.1186/s12913-020-05610-x (PMC7424658; doi:10.1186/s12913-020-05610-x)
Supplement: Supplementary file 1 — Additional file 1: Annex 1. Prepared form. Annex 2. Geographic Distribution of CT and MRI at public hospitals in all states and regions of Myanmar (2016). Annex 3. Geographic Distribution of CT and MRI at public hospitals in all states and regions of Myanmar (2015). Annex 4. Lorenz Curve of CT at public hospitals in all states and regions of Myanmar (2016). Annex 5. Lorenz Curve of CT at public hospitals in all states and regions of Myanmar (2015). Annex 6. Lorenz Curve of MRI at public hospitals in all states and regions of Myanmar (2016). Annex 7. Lorenz Curve of MRI at public hospitals in all states and regions of Myanmar (2015). Annex 8. Population coverage and utilisation of CT and MRI at public hospitals in all states and regions of Myanmar (2016). Annex 9. Population coverage and utilisation of CT and MRI at public hospitals in all states and regions of Myanmar (2015). [file 12913_2020_5610_MOESM1_ESM.docx]

**Annex 1 Prepared form**

**Public Hospitals with CT scan**

| SN | Region/State | Hospital | Quantity | Type | Budget Year | Remark |
| --- | --- | --- | --- | --- | --- | --- |
| 1 |  |  |  |  |  |  |
| 2 |  |  |  |  |  |  |
| 3 |  |  |  |  |  |  |
| 4 |  |  |  |  |  |  |

**Public Hospitals with MRI scan**

| SN | Region/State | Hospital | Quantity | Type | Budget Year | Remark |
| --- | --- | --- | --- | --- | --- | --- |
| 1 |  |  |  |  |  |  |
| 2 |  |  |  |  |  |  |
| 3 |  |  |  |  |  |  |
| 4 |  |  |  |  |  |  |

**CT Types of machines**

| SN | Hospital | State/ Region | Machine characteristics | | | |
| --- | --- | --- | --- | --- | --- | --- |
|  |  |  | Type | Number | Cost | Year of purchased/ used |
|  |  |  |  |  |  |  |
| 1 |  |  |  |  |  |  |
| 2 |  |  |  |  |  |  |
| 3 |  |  |  |  |  |  |
| 4 |  |  |  |  |  |  |

**MRI Types of machines**

| SN | Hospital | State/ Region | Machine characteristics | | | |
| --- | --- | --- | --- | --- | --- | --- |
|  |  |  | Type of MRI | Number | Cost | Year of Purchased |
|  |  |  |  |  |  |  |
| 1 |  |  |  |  |  |  |
| 2 |  |  |  |  |  |  |
| 3 |  |  |  |  |  |  |
| 4 |  |  |  |  |  |  |

**CT examination region**

| SN | Hospital | State/ Region | Year | CT Scan | | | | | | | | | | | | | | | | |
| --- | --- | --- | --- | --- | --- | --- | --- | --- | --- | --- | --- | --- | --- | --- | --- | --- | --- | --- | --- | --- |
|  |  |  |  | Examination Region | | | | | | | | | | | | | | | | |
|  |  |  |  | Head | Head & Neck | Head & other region | Whole spine | Cervical spine | Thoracic & thoraco- lumbar spine | Lumbar spine or lumbo- sacral spine | Abdomen &/or pelvic | Cervical | Nasal sinuses | Thorax | Body (Thorax and abdomen) | whole body | Angiogram | Limb/ Joint | Other (specify) | Total |
|  |  |  |  |  |  |  |  |  |  |  |  |  |  |  |  |  |  |  |  |  |
|  |  |  |  |  |  |  |  |  |  |  |  |  |  |  |  |  |  |  |  |  |

**MRI Examination region**

| SN | Hospital | State/ Region | Year | MRI | | | | | | | | | | | | | |
| --- | --- | --- | --- | --- | --- | --- | --- | --- | --- | --- | --- | --- | --- | --- | --- | --- | --- |
|  |  |  |  | Examination Region | | | | | | | | | | | | | |
|  |  |  |  | Brain/ Head | Whole spine | Cervical spine | Thoracic & Thoraco- lumbar spine | Lumbar spine | Abdomen&/or pelvic | Pelvic | Both hips | Thorax | Limbs/ Joints | MRCP | MRA / MRV | Other (Specify) | Total |
|  |  |  |  |  |  |  |  |  |  |  |  |  |  |  |  |  |  |
|  |  |  |  |  |  |  |  |  |  |  |  |  |  |  |  |  |  |
|  |  |  |  |  |  |  |  |  |  |  |  |  |  |  |  |  |  |

**CT Patient List**

| SN. | Hospital | State/ Region | Year | CT Examination | | | | | | | | | | | |
| --- | --- | --- | --- | --- | --- | --- | --- | --- | --- | --- | --- | --- | --- | --- | --- |
|  |  |  |  | Patients characteristics | | | | | | | | | | | |
|  |  |  |  | Total | Male | Female | Age | | | | | | | | |
|  |  |  |  |  |  |  | ≤ 15 | 16-25 | 26-35 | 36-45 | 46-55 | 56-65 | 66-75 | ≥ 76 | Total |
|  |  |  |  |  |  |  |  |  |  |  |  |  |  |  |  |
|  |  |  |  |  |  |  |  |  |  |  |  |  |  |  |  |
|  |  |  |  |  |  |  |  |  |  |  |  |  |  |  |  |
|  |  |  |  |  |  |  |  |  |  |  |  |  |  |  |  |
|  |  |  |  |  |  |  |  |  |  |  |  |  |  |  |  |

**MRI Patient List**

| SN | Hospital | State/ Region | Year | MRI Examination | | | | | | | | | | | |
| --- | --- | --- | --- | --- | --- | --- | --- | --- | --- | --- | --- | --- | --- | --- | --- |
|  |  |  |  | Patients characteristics | | | | | | | | | | | |
|  |  |  |  | Total | Male | Female |  | Age | | | | | | | |
|  |  |  |  |  |  |  | ≤ 15 | 16-25 | 26-35 | 36-45 | 46-55 | 56-65 | 66-75 | ≥ 76 | Total |
|  |  |  |  |  |  |  |  |  |  |  |  |  |  |  |  |
|  |  |  |  |  |  |  |  |  |  |  |  |  |  |  |  |
|  |  |  |  |  |  |  |  |  |  |  |  |  |  |  |  |
|  |  |  |  |  |  |  |  |  |  |  |  |  |  |  |  |
|  |  |  |  |  |  |  |  |  |  |  |  |  |  |  |  |

**Annex 2. Geographic Distribution of CT and MRI at public hospitals in all states and regions of Myanmar (2016)**

| **Region/State/**  **Union Territory** | **CT** | | | | | |  |  |  | **MRI** | | | | | | | |
| --- | --- | --- | --- | --- | --- | --- | --- | --- | --- | --- | --- | --- | --- | --- | --- | --- | --- |
|  | **Tertiary  hospital** |  | **Secondary  hospital** |  |  | **Total** |  | **%** |  | **Tertiary  hospital** |  | **Secondary  hospital** |  |  | **Total** |  | **%** |
| Kachin | - |  | 2 |  |  | 2 |  | 5.1 |  | - |  | - |  |  | - |  |  |
| Kayah | - |  | 1 |  |  | 1 |  | 2.6 |  | - |  | - |  |  | - |  |  |
| Kayin | - |  | 1 |  |  | 1 |  | 2.6 |  | - |  | - |  |  | - |  |  |
| Chin | - |  | 1 |  |  | 1 |  | 2.6 |  | - |  | - |  |  | - |  |  |
| Sagaing | - |  | 1 |  |  | 1 |  | 2.6 |  | - |  | - |  |  | - |  |  |
| Tanintharyi | - |  | 2 |  |  | 2 |  | 5.1 |  | - |  | - |  |  | - |  |  |
| Bago | - |  | 1 |  |  | 1 |  | 2.6 |  | - |  | - |  |  | - |  |  |
| Magway | - |  | 2 |  |  | 2 |  | 5.1 |  | - |  | 1 |  |  | 1 |  | 8.3 |
| Mandalay | 6 |  |  |  |  | 6 |  | 15.4 |  | 2 |  | - |  |  | 2 |  | 16.7 |
| Mon | - |  | 1 |  |  | 1 |  | 2.6 |  | - |  | - |  |  | - |  | - |
| Rakhine | - |  | 1 |  |  | 1 |  | 2.6 |  | - |  | - |  |  | - |  | - |
| Yangon | 13 |  | - |  |  | 13 |  | 33.2 |  | 6 |  | - |  |  | 6 |  | 50.0 |
| Shan | 1 |  | 2 |  |  | 3 |  | 7.7 |  | 1 |  | - |  |  | 1 |  | 8.3 |
| Ayeyarwady | - |  | 2 |  |  | 2 |  | 5.1 |  | - |  | - |  |  | 0 |  | 0 |
| Nay Pyi Taw | 2 |  | - |  |  | 2 |  | 5.1 |  | 2 |  | - |  |  | 2 |  | 16.7 |
| **Total** | **22** |  | **17** |  |  | **39** |  | **100** |  | **11** |  | **1** |  |  | **12** |  | **100** |

**Annex 3. Geographic Distribution of CT and MRI at public hospitals in all states and regions of Myanmar (2015)**

| **Region/State/**  **Union Territory** | **CT** | | | | | |  |  |  | **MRI** | | | | | | | |
| --- | --- | --- | --- | --- | --- | --- | --- | --- | --- | --- | --- | --- | --- | --- | --- | --- | --- |
|  | **Tertiary  hospital** |  | **Secondary  hospital** |  |  | **Total** |  | **%** |  | **Tertiary  hospital** |  | **Secondary  hospital** |  |  | **Total** |  | **%** |
| Kachin | - |  | 1 |  |  | 1 |  | 3.2 |  | - |  | - |  |  | - |  |  |
| Kayah | - |  | 1 |  |  | 1 |  | 3.2 |  | - |  | - |  |  | - |  |  |
| Kayin | - |  | 1 |  |  | 1 |  | 3.2 |  | - |  | - |  |  | - |  |  |
| Chin | - |  | 0 |  |  | 0 |  | 0 |  | - |  | - |  |  | - |  |  |
| Sagaing | - |  | 0 |  |  | 0 |  | 0 |  | - |  | - |  |  | - |  |  |
| Tanintharyi | - |  | 2 |  |  | 2 |  | 6.5 |  | - |  | - |  |  | - |  |  |
| Bago | - |  | 1 |  |  | 1 |  | 3.2 |  | - |  | - |  |  | - |  |  |
| Magway | - |  | 2 |  |  | 2 |  | 6.5 |  | - |  | - |  |  | - |  |  |
| Mandalay | 5 |  | - |  |  | 5 |  | 16.1 |  | 2 |  | - |  |  | 2 |  | 28.6 |
| Mon | - |  | 1 |  |  | 1 |  | 3.2 |  | - |  | - |  |  | - |  | 0.0 |
| Rakhine | - |  | 1 |  |  | 1 |  | 3.2 |  | - |  | - |  |  | - |  | 0.0 |
| Yangon | 11 |  | - |  |  | 11 |  | 35.5 |  | 3 |  | - |  |  | 3 |  | 42.9 |
| Shan | 1 |  | 1 |  |  | 2 |  | 6.5 |  | 0 |  | - |  |  |  |  |  |
| Ayeyarwady | - |  | 1 |  |  | 1 |  | 3.2 |  | - |  | - |  |  | - |  | 0.0 |
| Nay Pyi Taw | 2 |  | - |  |  | 2 |  | 6.5 |  | 2 |  | - |  |  | 2 |  | 28.5 |
| **Total** | **19** |  | **12** |  |  | **31** |  | **100** |  | **7** |  |  |  |  | **7** |  | **100** |

**Annex 4. Lorenz Curve of CT at public hospitals in all states and regions of Myanmar (2016)**

**Gini coefficient = 0.41**

**Annex 5. Lorenz Curve of CT at public hospitals in all states and regions of Myanmar (2015)**

**Gini coefficient = 0.47**

**Annex 6. Lorenz Curve of MRI at public hospitals in all states and regions of Myanmar (2016)**

**Gini coefficient = 0.70**

**Annex 7. Lorenz Curve of MRI at public hospitals in all states and regions of Myanmar (2015)**

**Gini coefficient = 0.78**

**Annex 8. Population coverage and utilisation of CT and MRI at public hospitals in all states and regions of Myanmar (2016)**

| **Region/State/**  **Union Territory** | **Population** | **CT** | | | |  | **MRI** | | | |
| --- | --- | --- | --- | --- | --- | --- | --- | --- | --- | --- |
|  |  | **Number** | **Coverage per million population** | **No. of examination** | **Examination per 1,000 population** |  | **Number** | **Coverage per million population** | **No. of examination** | **Examination per 1,000 population** |
| Kachin | 1,796,122 | 2 | 1.11 | 563 | 0.31 |  | 0 | - | - | - |
| Kayah | 303,646 | 1 | 3.29 | 490 | 1.61 |  | 0 | - | - | - |
| Kayin | 1,584,498 | 1 | 0.63 | 890 | 0.56 |  | 0 | - | - | - |
| Chin | 502,705 | 1 | 1.99 | 283 | 0.56 |  | 0 | - | - | - |
| Sagaing | 5,451,343 | 1 | 0.18 | 142 | 0.03 |  | 0 | - | - | - |
| Tanintharyi | 1,447,293 | 2 | 1.38 | 1,952 | 1.35 |  | 0 | - | - | - |
| Bago | 4,907,824 | 1 | 0.20 | 840 | 0.17 |  | 0 | - | - | - |
| Magway | 3,943,235 | 2 | 0.51 | 2,054 | 0.52 |  | 1 | 0.25 | 25 | 0.01 |
| Mandalay | 6,331,301 | 6 | 0.95 | 3,811 | 0.60 |  | 2 | 0.32 | 544 | 0.09 |
| Mon | 2,022,755 | 1 | 0.49 | 1,732 | 0.86 |  | 0 | - |  | - |
| Rakhine | 3,283,218 | 1 | 0.30 | 676 | 0.21 |  | 0 | - |  | - |
| Yangon | 7,763,687 | 13 | 1.67 | 44,845 | 5.78 |  | 6 | 0.77 | 4,121 | 0.53 |
| Shan | 6,094,321 | 3 | 0.49 | 1,136 | 0.19 |  | 1 | 0.16 | 74 | 0.01 |
| Ayeyarwady | 6,267,418 | 2 | 0.32 | 881 | 0.14 |  | 0 | - |  | - |
| Nay Pyi Taw | 1,217,325 | 2 | 1.64 | 7,666 | 6.30 |  | 2 | 1.64 | 1,318 | 1.08 |
| **Total** | **52,916,691** | **39** | **0.74** | **67,961** | **1.28** |  | **12** | **0.23** | **6,082** | **0.11** |

**Annex 9. Population coverage and utilisation of CT and MRI at public hospitals in all states and regions of Myanmar (2015)**

| **Region/State/**  **Union Territory** | **Population** | **CT** | | | |  | **MRI** | | | |
| --- | --- | --- | --- | --- | --- | --- | --- | --- | --- | --- |
|  |  | **Number** | **Coverage per million population** | **No. of examination** | **Examination per 1,000 population** |  | **Number** | **Coverage per million population** | **No. of examination** | **Examination per 1,000 population** |
| Kachin | 1,762,901 | 1 | 0.57 | 518 | 0.29 |  | 0 | - | - | - |
| Kayah | 297,162 | 1 | 3.37 | 444 | 1.49 |  | 0 | - | - | - |
| Kayin | 1,575,826 | 1 | 0.63 | 639 | 0.41 |  | 0 | - | - | - |
| Chin | 497,009 | 0 | 0 | 0 | 0.00 |  | 0 | - | - | - |
| Sagaing | 5,411,440 | 0 | 0 | 0 | 0.00 |  | 0 | - | - | - |
| Tanintharyi | 1,434,723 | 2 | 1.39 | 1,347 | 0.94 |  | 0 | - | - | - |
| Bago | 4,896,520 | 1 | 0.20 | 581 | 0.12 |  | 0 | - | - | - |
| Magway | 3,944,972 | 2 | 0.51 | 1,729 | 0.44 |  | 0 | - | - | - |
| Mandalay | 6,274,139 | 5 | 0.80 | 2,464 | 0.39 |  | 2 | 0.32 | 645 | 0.10 |
| Mon | 2,034,439 | 1 | 0.49 | 883 | 0.43 |  | 0 | - |  | - |
| Rakhine | 3,266,405 | 1 | 0.31 | 146 | 0.04 |  | 0 | - |  | - |
| Yangon | 7,595,018 | 11 | 1.45 | 32,633 | 4.30 |  | 3 | 0.39 | 4,759 | 0.63 |
| Shan | 6,001,082 | 2 | 0.33 | 656 | 0.11 |  | 0 | - | - | - |
| Ayeyarwady | 6,262,164 | 1 | 0.16 | 578 | 0.09 |  | 0 | - | - | - |
| Nay Pyi Taw | 1,196,717 | 2 | 1.67 | 6,002 | 5.02 |  | 2 | 1.67 | 716 | 0.60 |
| **Total** | **52,450,517** | **31** | **0.59** | **48,620** | **0.93** |  | **7** | **0.13** | **6,082** | **0.12** |
